# Supplementary material for: The dynamics of SARS-CoV-2 infectivity with changes in aerosol microenvironment
Source: Proc Natl Acad Sci U S A. 2022 Jun 28;119(27):e2200109119. doi: 10.1073/pnas.2200109119 (PMC9271203; doi:10.1073/pnas.2200109119)
Supplement: Supplementary File [file pnas.2200109119.sapp.pdf]

The Dynamics of SARS-CoV-2 Infectivity with Changes in Aerosol Microenvironment - Supplementary Information

Henry P. Oswin[1], Allen E. Haddrell[1]*, Mara Otero-Fernandez[1], Jamie F.S. Mann[2], Tristan A. Cogan[2], Thomas G. Hilditch[1], Jianghan Tian[1], Daniel A. Hardy[1], Darryl J. Hill[3], Adam Finn[3], Andrew D. Davidson[3]*, and Jonathan P. Reid[1]*

[1]School of Chemistry, Cantock's Close, University of Bristol, Bristol, UK

[2]Bristol Veterinary School, University of Bristol, Langford House, Langford, Bristol, UK

[3]School of Cellular and Molecular Medicine, University of Bristol, Bristol, UK

Extended Materials and Methods

Virus Strains and Culture Methods

Vero E6 cells modified to constitutively express TMPRSS2 (Vero E6/TMPRSS2 cells (1); obtained from NIBSC, UK) were cultured at 37°C and 5% CO_2 in Dulbecco's Modified Eagle Medium (DMEM, high glucose; Sigma, UK) supplemented with 10% foetal bovine serum (FBS, Sigma), 100 units/ml penicillin (Gibco, UK), 100 µg/ml streptomycin (Gibco, UK), and L-glutamine (Gibco, UK). The SARS-CoV-2 viral isolates, SARS-CoV-2/human/Liverpool/REMRQ0001/2020 (REMRQ0001, GenBank: MW041156.1), hCoV-19/England/02/2020 (GISAID ID: EPI_ISL_407073) and the "Bristol" variant derived from it (BriSΔ) in which spike amino acids 679-687 (NSPRRARSV) had been deleted and replaced with Ile (2) were isolated as previously described (3). The SARS-CoV-2 variants hCoV-19/England/204690005/2020 (lineage B.1.1.7 - Alpha variant; GISAID ID: EPI_ISL_693401) and hCoV-19/England/205280030/2020 (lineage B.1.351 - Beta variant; GISAID ID: EPI_ISL_770441) were kindly provided by Professor Wendy Barclay, Imperial College, London and Professor Maria Zambon, Public Health England. Stocks of SARS-CoV-2 isolates were produced by inoculation of Vero E6/TMPRSS2 cells at a multiplicity of infection (MOI) of 0.01 and incubating the cells for 48-72 h in Eagle's minimum essential medium plus GlutaMAX (MEM, Gibco, ThermoFisher, cat# 41090036) supplemented with 2% v/v FBS and 0.1 mM non-essential amino acids (MEM 2% FBS). The culture supernatants were clarified by filtration through a 0.2 µM filter and stored in aliquots at − 80 °C. The titre of the stocks was determined by preparing 10-fold serial dilutions in MEM 2% FBS which were added to 1 × 10^4 Vero E6 cells in the same medium in each of 12 wells of a 96-well plate. Plates were incubated at 37 °C for 4 - 7 days and then examined for cytopathic effect (CPE). The $TCID_{50}$ was calculated according to the method of Reed and Muench (4).

Quantification of Virus

SARS-CoV-2 in aerosol experiments was quantified by measuring the occurrence of viral induced CPE on Vero E6/TMPRSS2 cells seeded in 96-well plates. Viral stock titres were calculated by $TCID_{50}$. A 10-fold dilution series of the virus suspension was prepared in DMEM 2% FBS and each dilution was then used to infect a row of cells with 100µl of virus. The plate was incubated for 4 - 6 days at 37°C 5% CO_2 and CPE then observed by microscopy. By counting the number of infected wells on each row the virus titre can be calculated using the Reed-Muench method (4).

When the virus concentration was lower than 10^2 infectious units ml^{-1}, as was the case for levitated virus, an alternative approach was used. The entire neat suspension was used to inoculate Vero E6/TMPRSS2 cell seeded wells in a 96 well plate with 100µl of sample per well. Depending on the volume of the sample, the number of wells inoculated would vary, but typically the SARS-CoV-2 levitations would be deposited into 6 ml of media and then used to inoculate 60 wells in a plate, such that the outermost wells of the plate were left empty. The plate was then incubated for 4 - 6 days at 37°C and the cytopathy assessed by microscopy. To calculate the amount of virus in the plate, a rearranged form of the Poisson distribution equation was used:

$$-\ln P(X = 0) = \lambda$$

Where *P(X=0)* is the proportion of uninfected wells (calculated by dividing the total number of uninfected wells by the total number of inoculated wells) and λ is the calculated infectious units per 100µl. By multiplying λ by the total volume of the sample and then dividing it by the number of droplets deposited into the sample, the number of infectious units per droplet can be calculated.

Bulk Stability Measurements

To assess the stability of SARS-CoV-2 in bulk solutions, the virus stock was first diluted to 10^{-4} in the test solution (DMEM or MEM with altered pH or solute concentration). This 10^{-4} solution was then incubated in a sealed tube at room temperature for 20 minutes. 100 µl was taken from this and diluted back into 900 µl of normal DMEM 2% FBS. 500 µl was then added to 19.5 ml of DMEM 2% FBS (when inoculating 3 sets of 60 wells). This final dilution (a concentration of $10^{-6.3}$ of the original stock) was then used to infect cells for the quantification of remaining viral infectivity by the same method used to quantify virus post levitation. This $10^{-6.3}$ dilution was chosen as the SARS-CoV-2 stocks typically have a $TCID_{50}$ of around 10^7 ml^{-1} meaning that a $10^{-6.3}$ dilution would result in around 50% of wells being infected granting accurate viral quantification. This dilution can be adjusted for different stock concentrations. A portion of the stock was also immediately diluted to $10^{-6.3}$ and used to infect cells to calculate a T0 control value to which the incubated sample can be normalised.

CK-EDB Measurements of MEM

The droplets were generated and trapped as described above. Once confined, approximately 100 ms after droplet formation, the droplet was illuminated by a 532 nm laser (Laser Quantum, Ventus continuous wave [CW]). A nitrogen gas flow of 200 mL/min at a temperature of 20 °C and a set RH (range from ~0% to >90%) was passed directly over the droplet. As the droplet changes size, the electrodynamic field was manipulated to account for these changes and ensure that it remained confined within the centre of both the trap and laser beam.

A CCD camera (Thorlabs) collected the light scattered from the droplet in the near-forward direction at a central scattering angle of 45°. Images of the phase function, with an angular range from 32° to 58°, were collected every ~10 ms providing high time resolution measurements of particle size and morphology. This range was selected for two reasons: it is readily accessible to numerous other individual droplet analysis devices and the central viewing angle of 45° allowed access to the region of the near-forward scattering that corresponds to the range of applicability of the geometric optics approximation (up to 60°) for particle sizing.(5)

When a droplet was spherical and homogeneous, its radius and refractive index of the droplet could be estimated (using a prescribed relationship between droplet radius and refractive index(6)) by fitting a collection of time dependent phase functions with a library of Mie theory simulations (as seen in Figures 3b and 4b). This method is computationally demanding. Alternatively, the absolute radius of the droplet could be inferred from the average angular difference between the maxima within the phase function using the geometrical optics approximation.(5) This approach allowed for rapid analysis of each collected frame in real-time.

Falling Droplet Column Imaging of MEM

See Hardy *et al* 2021 (7) for detailed methodology. Droplets of MEM 2% FBS were dispensed into an enclosed columnar chamber maintained at a constant RH by a flow of temperature and humidity-controlled air. The in-flight images were collected by a CCD camera with droplets illuminated by stroboscopic lighting from a high-power LED. Particles were collected at the bottom of the column and imaged by SEM.

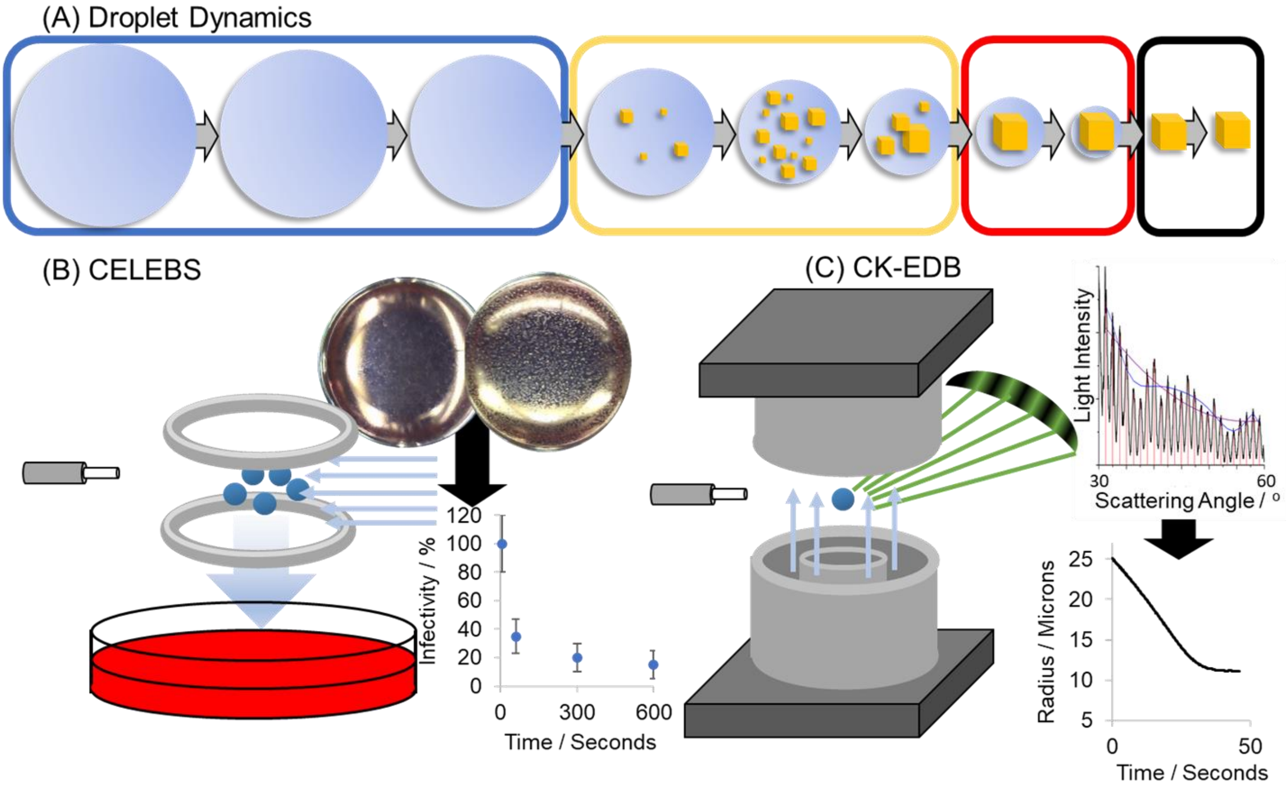

Figure S1| Experimental Approach. (A) Schematic of the physical changes that take place when an airborne aqueous droplet equilibrates to the surrounding relative humidity. On the left (blue box) the particle is an aqueous homogenous sphere. As the particle equilibrates to a sub-saturated RH, low-solubility solutes can precipitate inclusions within the droplet (yellow box). At a sufficiently low RH, the dominant solute (NaCl) can crystallise causing the particle to effloresce (black box). (B) Schematic of the CELEBS technique. Virus containing particles are levitated under controlled conditions and then deposited into media which is then plated onto a cell culture. By enumerating the cytopathic effect from that deposition, the amount of virus present can be quantified. (C) Schematic of the CK-EDB technique. Particles generated by the same droplet dispensers used in CELEBS are levitated under controlled conditions in the path of a laser. The physical changes that take place in that particle are studied through analysis of the light scattered by that particle.

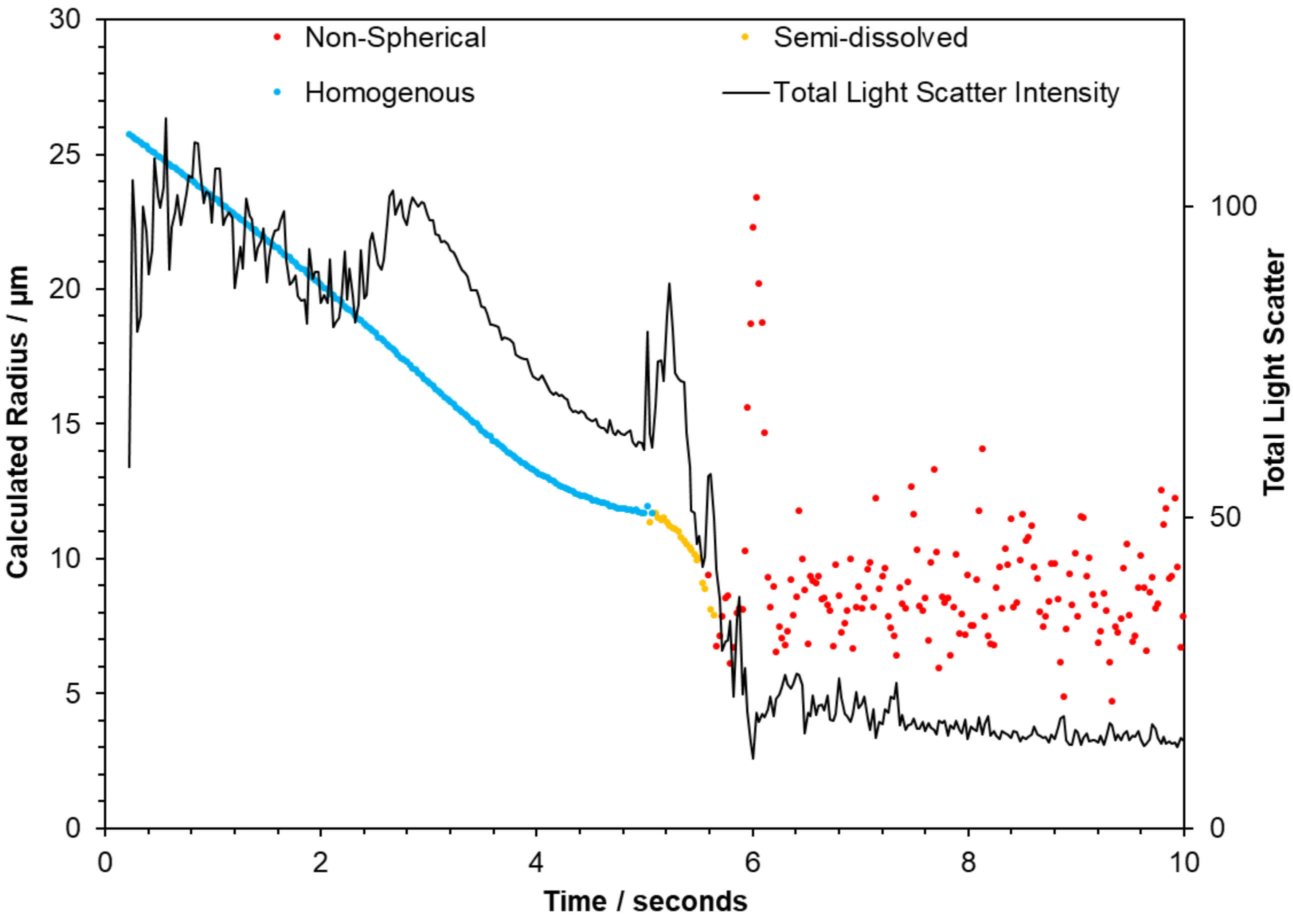

Figure S2| Sodium Chloride Efflorescence. A droplet of sodium chloride was trapped in the CK-EDB at 30% relative humidity (RH) and allowed to effloresce. The radius (plotted against the left y-axis) and structure was measured using the Mie scatter and is plotted in the coloured points. Blue points indicate a spherical homogenous droplet, yellow points indicate inclusions, and red points indicate a non-spherical particle (note that accurate sizing is not possible for non-spherical particles). The total light scatter intensity is plotted against the right-hand y-axis as a black line. The abrupt drop in total light scatter coincides with the efflorescence of the droplet.

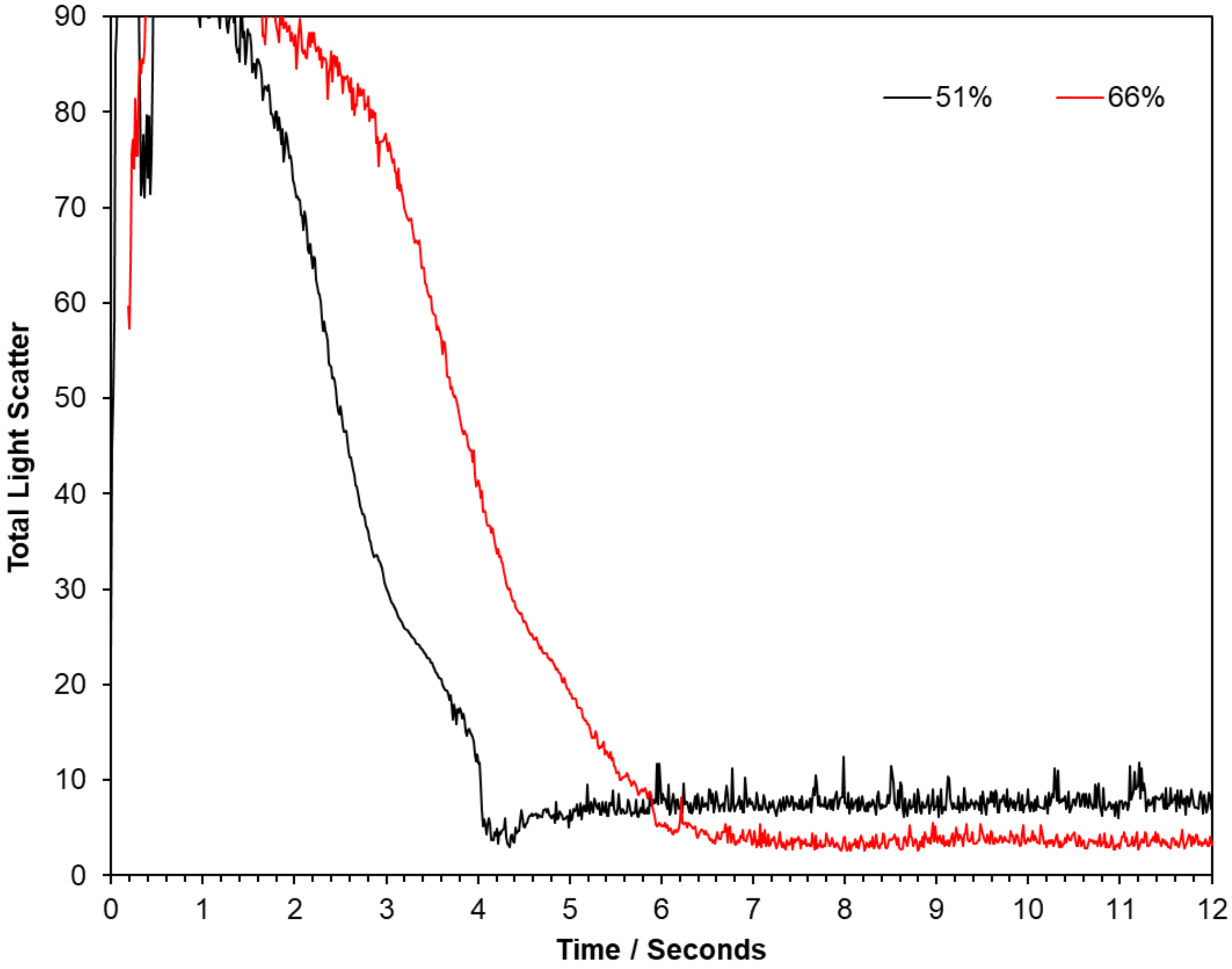

Figure S3| MEM 2% FBS Efflorescence. The total light scatter intensity was measured in the CK-EDB for droplets of MEM 2% FBS at a range of relative humidities. Shown here are the measurements at 51% RH (black line) and 66% RH (red line). At 51% RH the sudden drop in light intensity characteristic of efflorescence is observed. At 66% RH no efflorescence is observed.

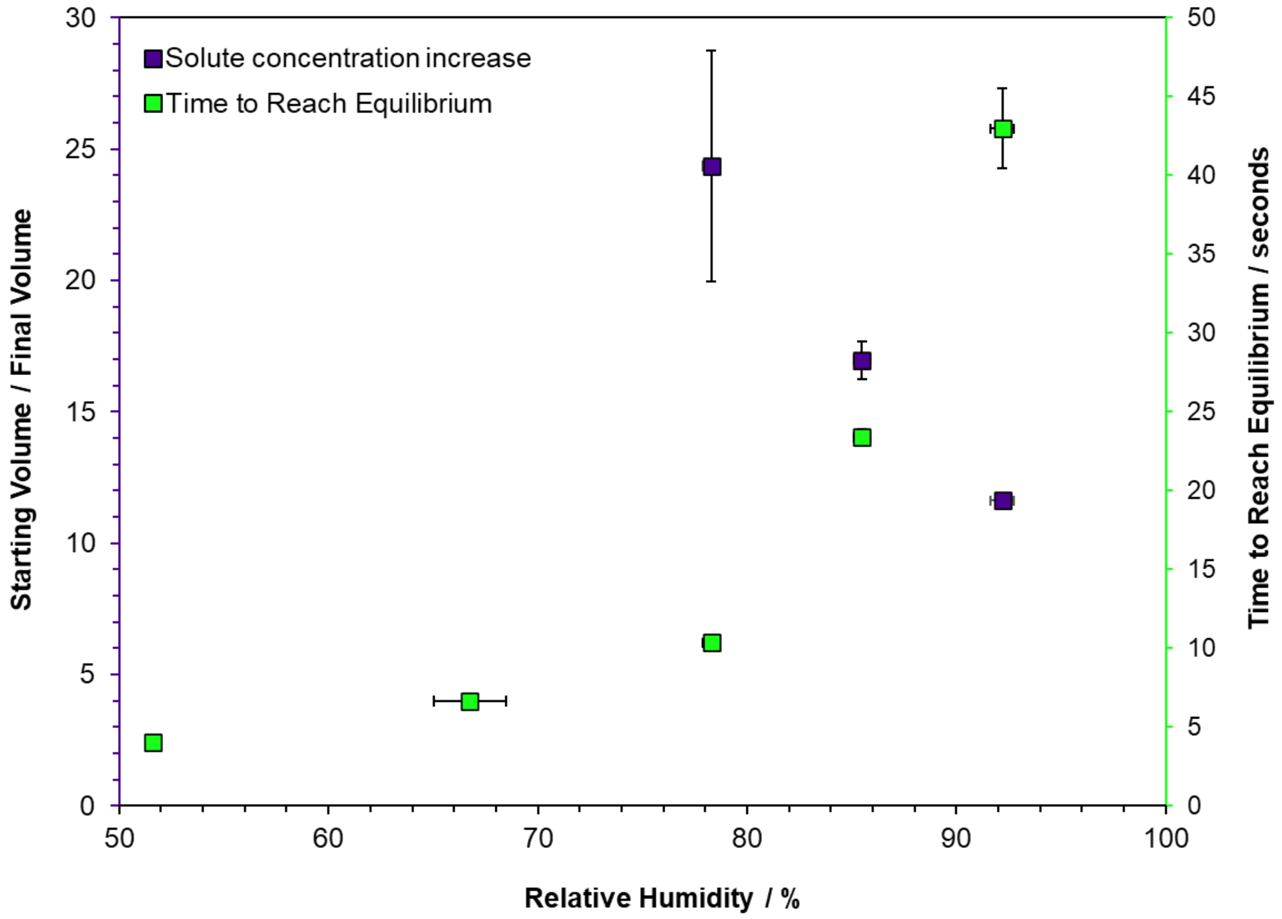

Figure S4| MEM 2% FBS Airborne Size Change. The ratio of initial volume of the droplet to the final volume droplet (purple, left-hand y-axis) and the time taken for the droplet to equilibrate (green, right-hand y-axis). Datapoints are the mean of 10 measurements. Error bars show the standard deviation of the RH (x error bars) and measurements (y error bars).

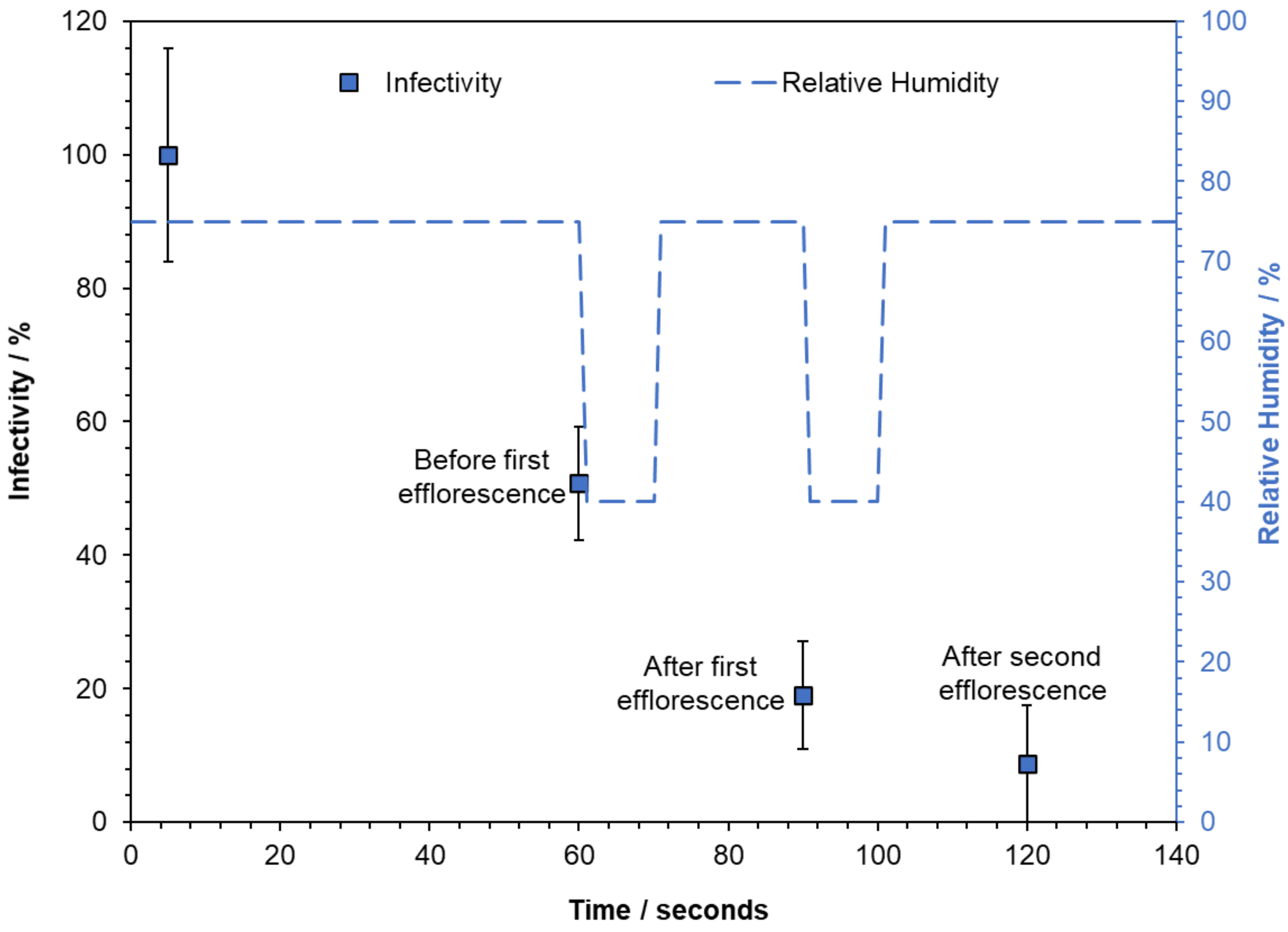

Figure S5| Infectivity loss driven by efflorescence. Infectivity curve for SARS-CoV-2 (REMRQ0001) levitated at fluctuating RH. The blue dotted line plotted against the left-hand y-axis shows the changes in the set RH throughout the experiment, whilst the blue datapoints show the % infectivity (mean of 4 measurements with error bars showing the standard error). Annotations have been added to blue datapoints for clarity.

Efflorescence driven loss of infectivity

The initial levitation at a RH above efflorescence (~75%) for 60 seconds resulted in a reduction in infectivity to ~50%. On a separate population of droplets, the RH was maintained at 75% for 60 seconds, but then transiently reduced from 75% to 40% for a period of 10 s, sufficient time to drive efflorescence, before being raised to 75% and the droplets collected. The infectivity at this point had fallen to ~19%. This was repeated but with two efflorescence cycles, reducing the RH to 40% a second time, and then raising it before sampling the droplets, resulting in an average infectivity of ~9%. In both instances of efflorescence, the infectivity fell to 35-50% of the preceding value, which is similar to the rapid loss seen at 40% RH (Fig. 1A).

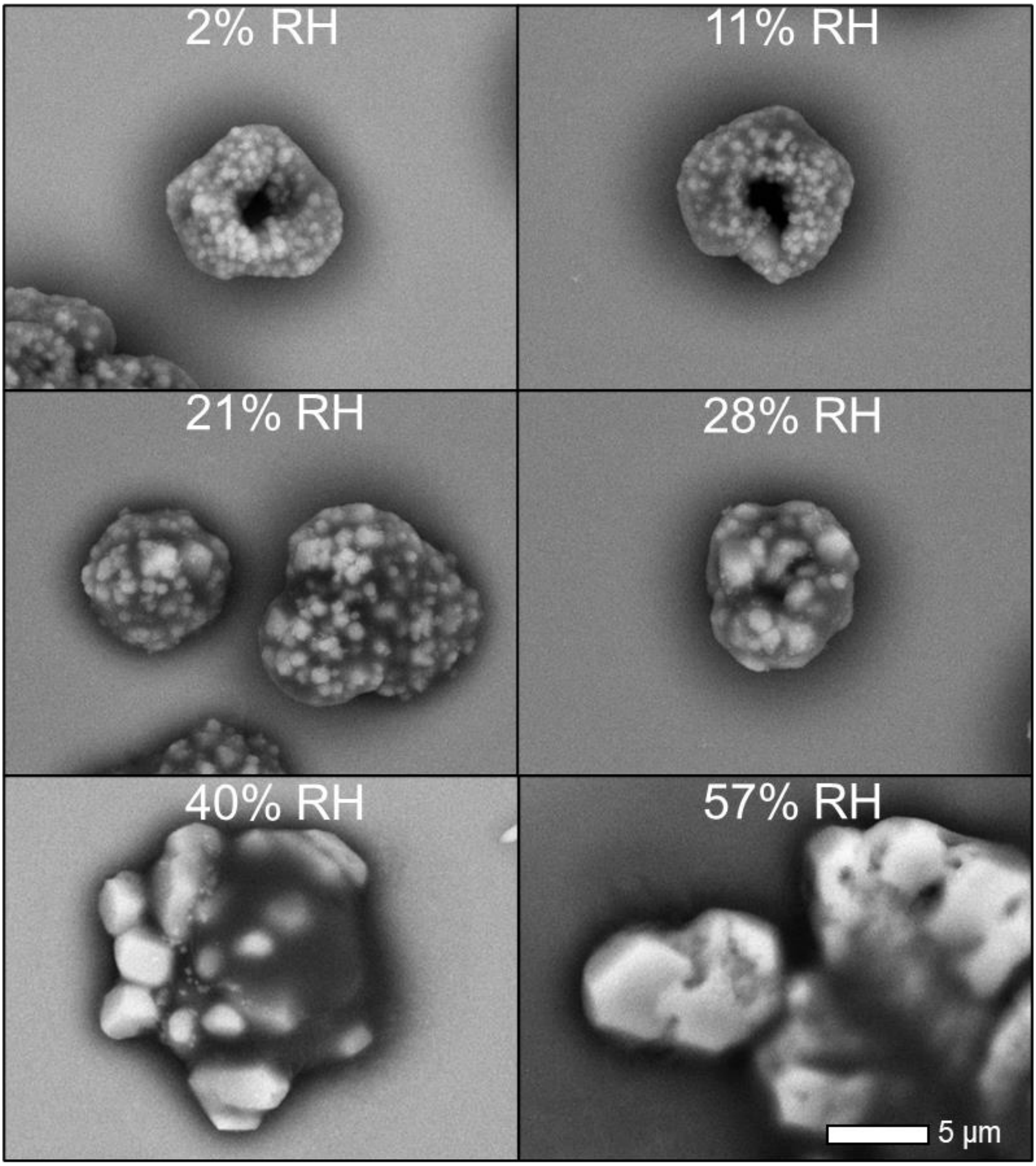

Figure S6| MEM 2% FBS SEM Images. SEM micrographs of MEM 2% FBS particles collected from the bottom of the falling droplet column after falling at a range of RHs. Note that the more spread-out salt crystals at 57% RH are likely the result of the particles still being liquid upon deposition. The scale bar (white at the bottom) show 5 µm.

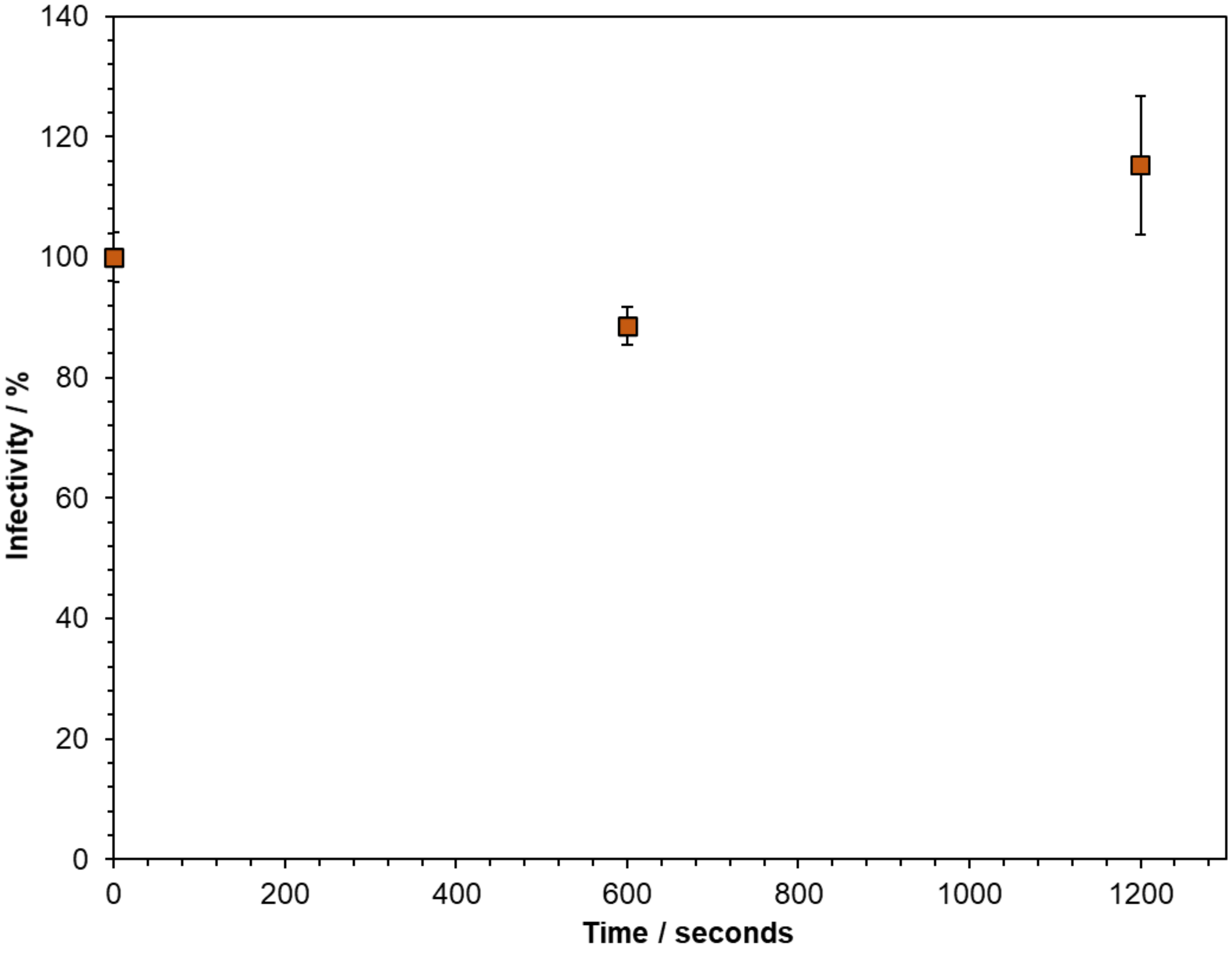

Figure S7| Bulk Survival of SARS-CoV-2 in 10x MEM. Bulk % infectivity measurement of SARS-CoV-2 (REMRQ0001) incubated for 20 minutes in 10x MEM 2% FBS before being diluted back into normal media (DMEM 10% FBS) and plated onto cells. Datapoints are the mean of 6 measurements for 10 minutes and 3 measurements for 20 minutes with error bars showing the standard error.

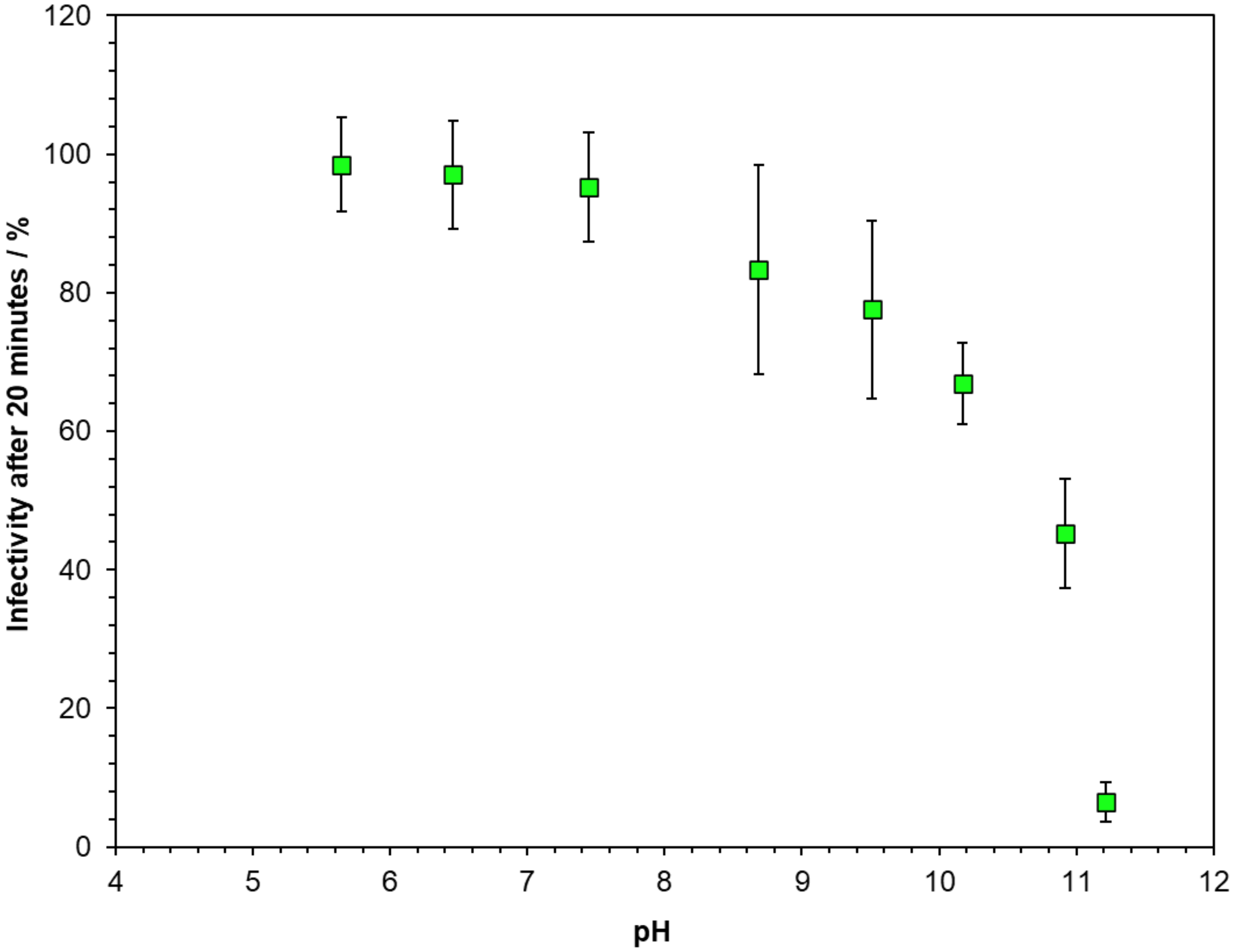

Figure S8| Infectivity loss of SARS-CoV-2 after high pH exposure. Bulk % infectivity of SARS-CoV-2 (B.1.1.7) after a 20-minute incubation in DMEM 2% FBS altered to a range of pHs, before being diluted back into neutral media and plated onto cells. Datapoints are the mean of ≥3 measurements with error bars showing the standard error.

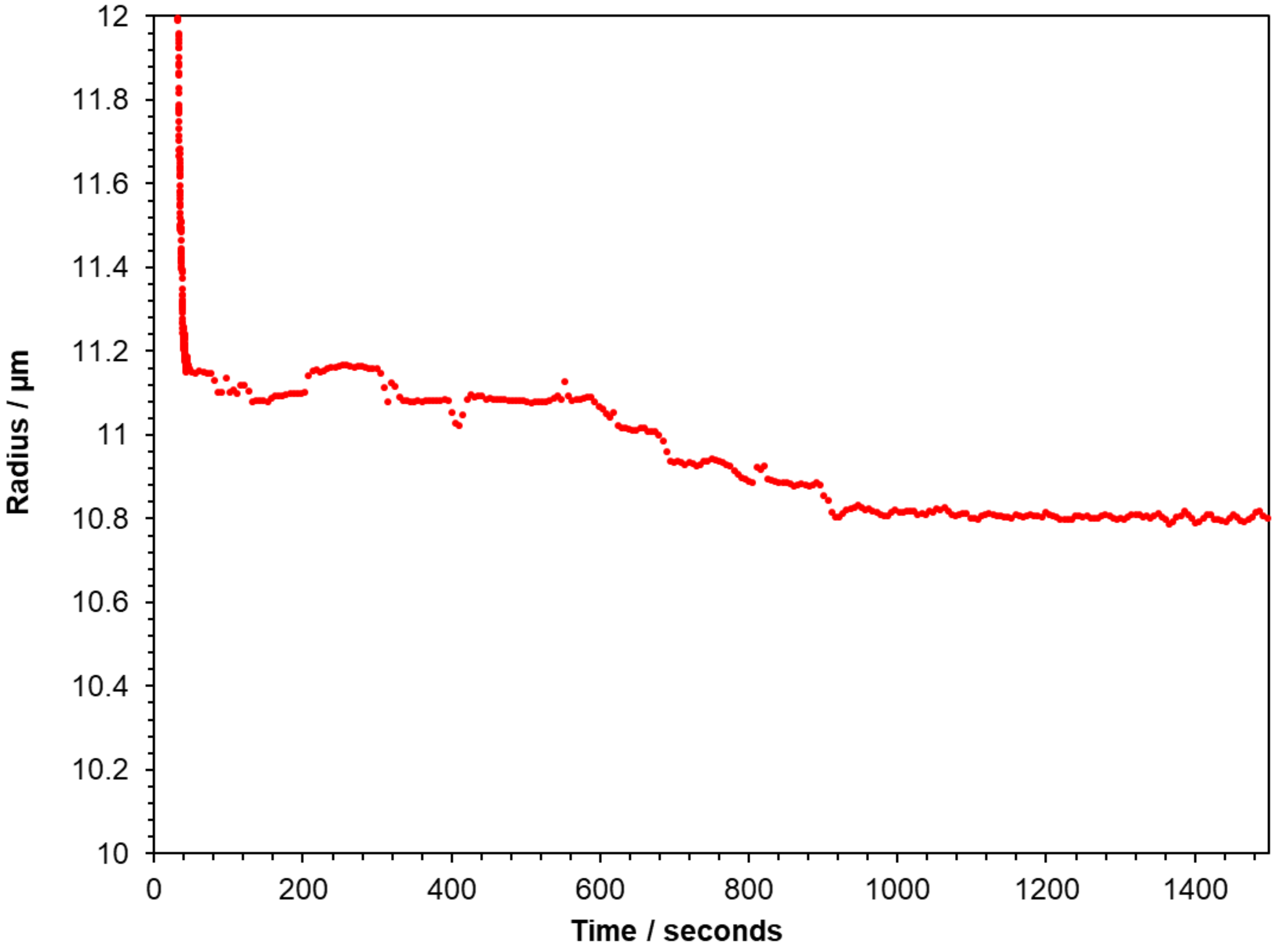

Figure S9| Change in MEM droplet radius as a result of CO_2 loss. CK-EDB measurement of an airborne MEM droplet shows the size continuing to decrease after the water equilibration is complete. The initial droplet radius was ~25 µm and the RH was ~90%. The initial size loss of the droplet finished after ~55 seconds as expected for a culture media levitation at high RH. However, the size of the droplet continued to slowly decrease for approximately 15 minutes after this initial size loss, indicating an additional component leaving the droplet.

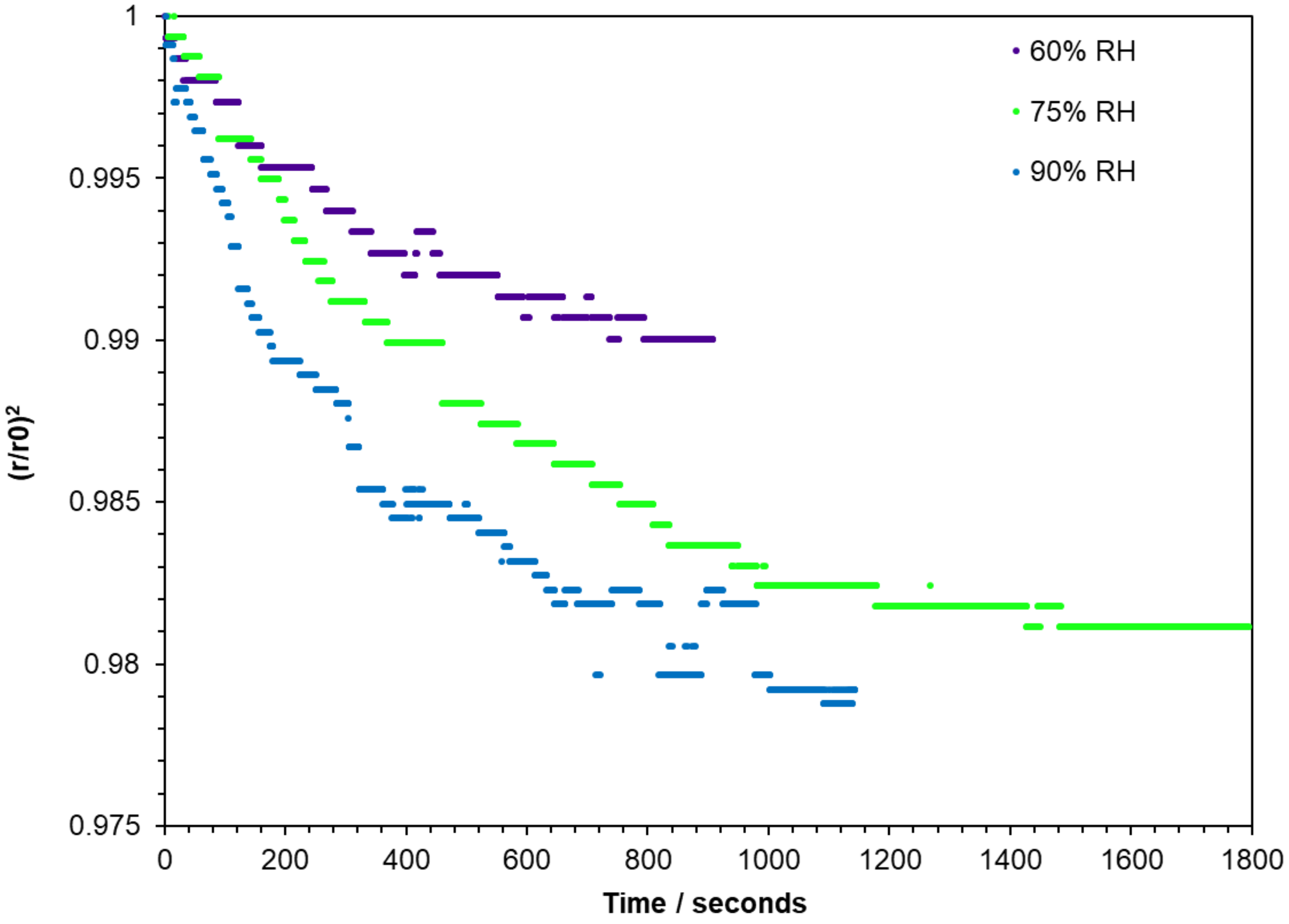

Figure S10| CO_2 loss from sodium bicarbonate droplets. CO_2 loss driven size change in airborne droplets. The evaporation of droplets of a 2:1 mass ratio of NaCl-NaHCO3 (to a final MFS of 0.08) was measured in the CK-EDB at 60% RH (purple), 75% RH (green) and 90% RH (blue). Radius is normalised to a point after the water has evaporated, allowing comparison of the radius change caused by CO_2 loss.

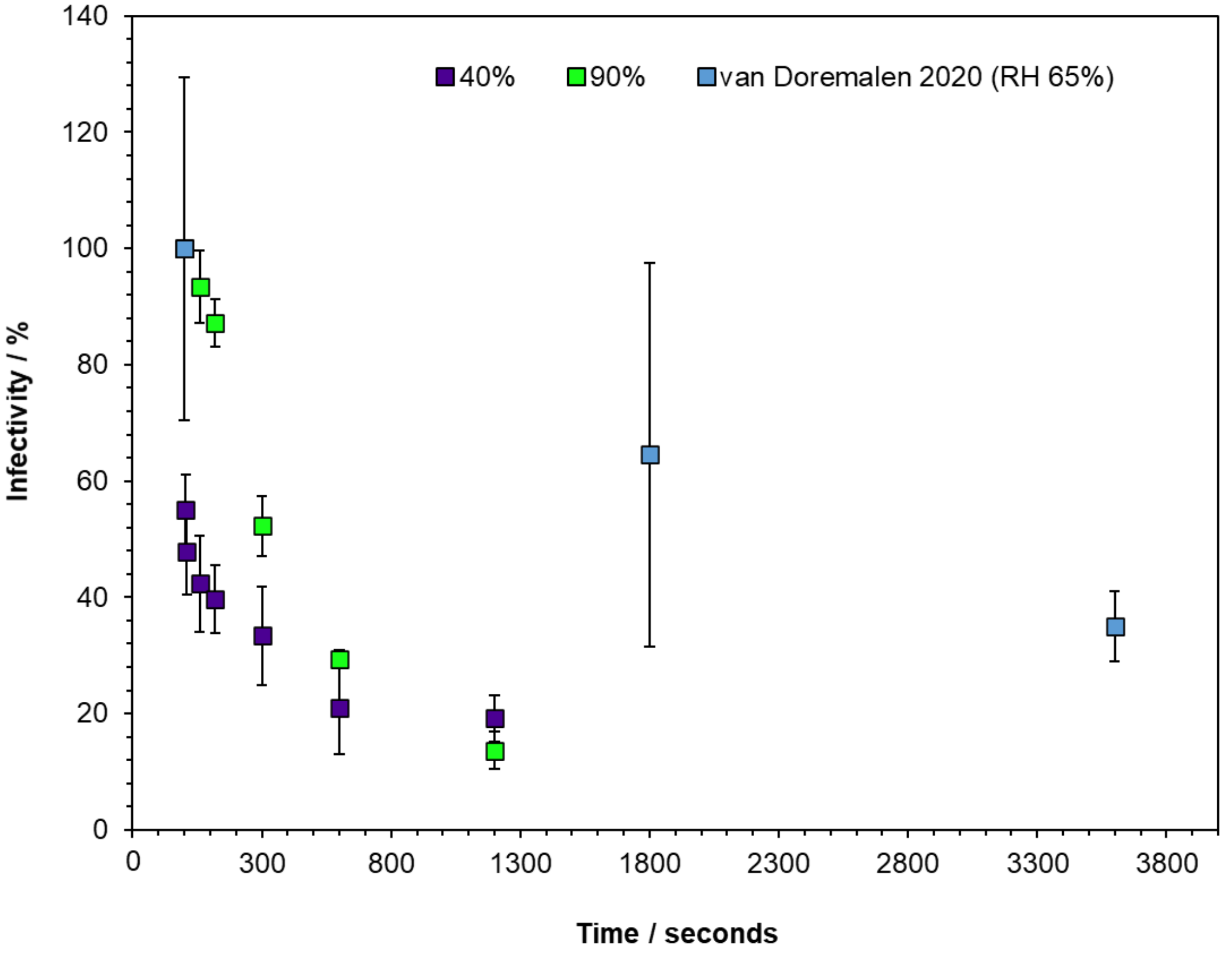

Figure S11| Comparison of CELEBS measurements to rotating drum measurement of SARS-CoV-2. Data from Figure 1a plotted alongside the SARS-CoV-2 airborne stability curve published by van Doremalen et al. Data from van Doremalen was normalised to their earliest measurement to allow direct comparison to the CELEBS data. CELEBS data plotted in purple and green is as described in Figure 1. Data from van Doremalen et al is plotted in blue with datapoints showing the mean of 3 measurements each and error bars showing the standard error.

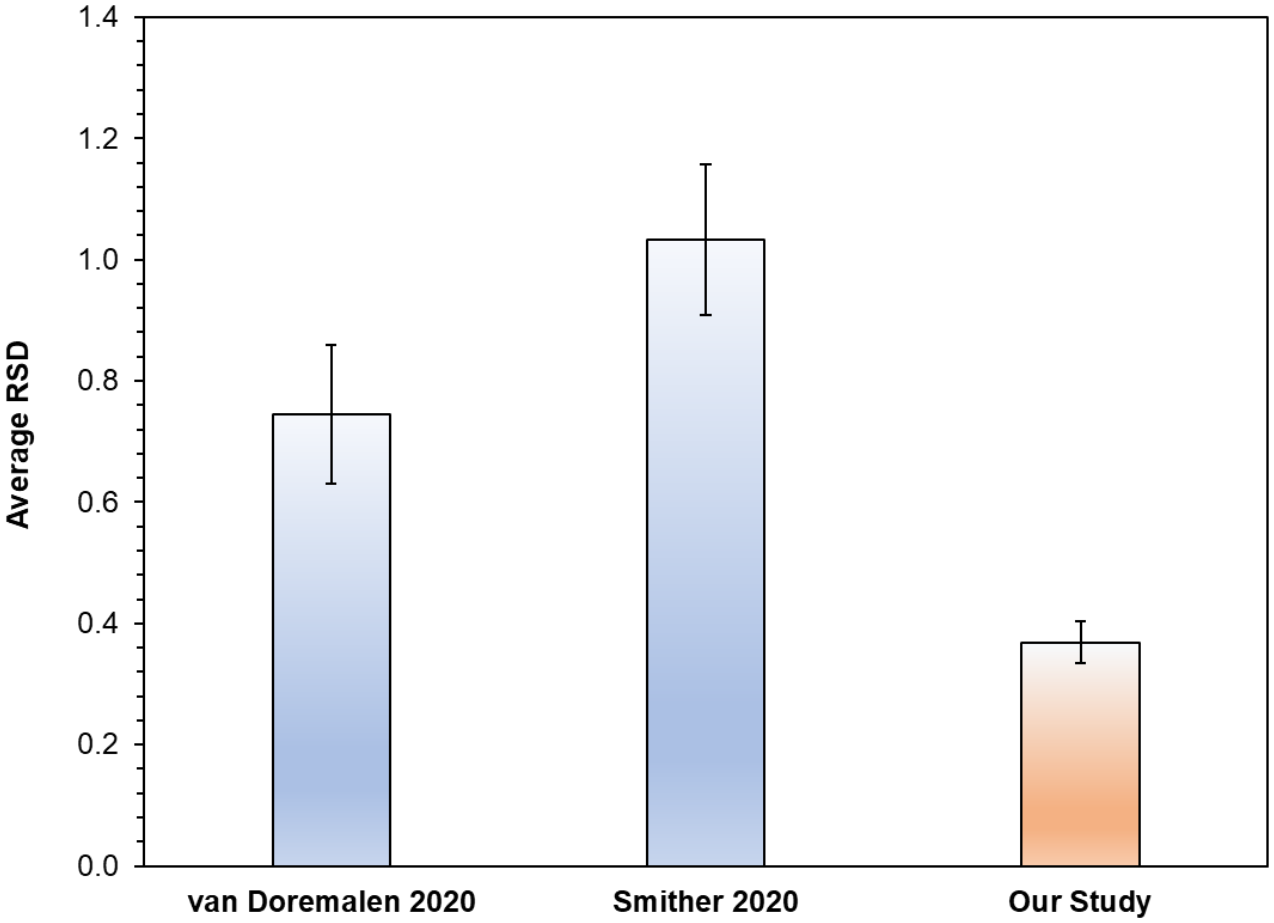

Figure S12| Comparison of CELEBS precision to rotating drum precision in measurements of SARS-CoV-2. Bar chart showing the RSDs calculated from SARS-CoV-2 aerostability datasets published by van Doremalen et al, Smither et al, and from this study. RSDs were calculated by dividing the standard deviation by the mean for each datapoint published. The average and standard error of these RSDs was then calculated and plotted. RSDs are the mean of 5 RSDs from van Doremalen 2020, 17 RSDs from Smither 2020, and 19 RSDs from our study.

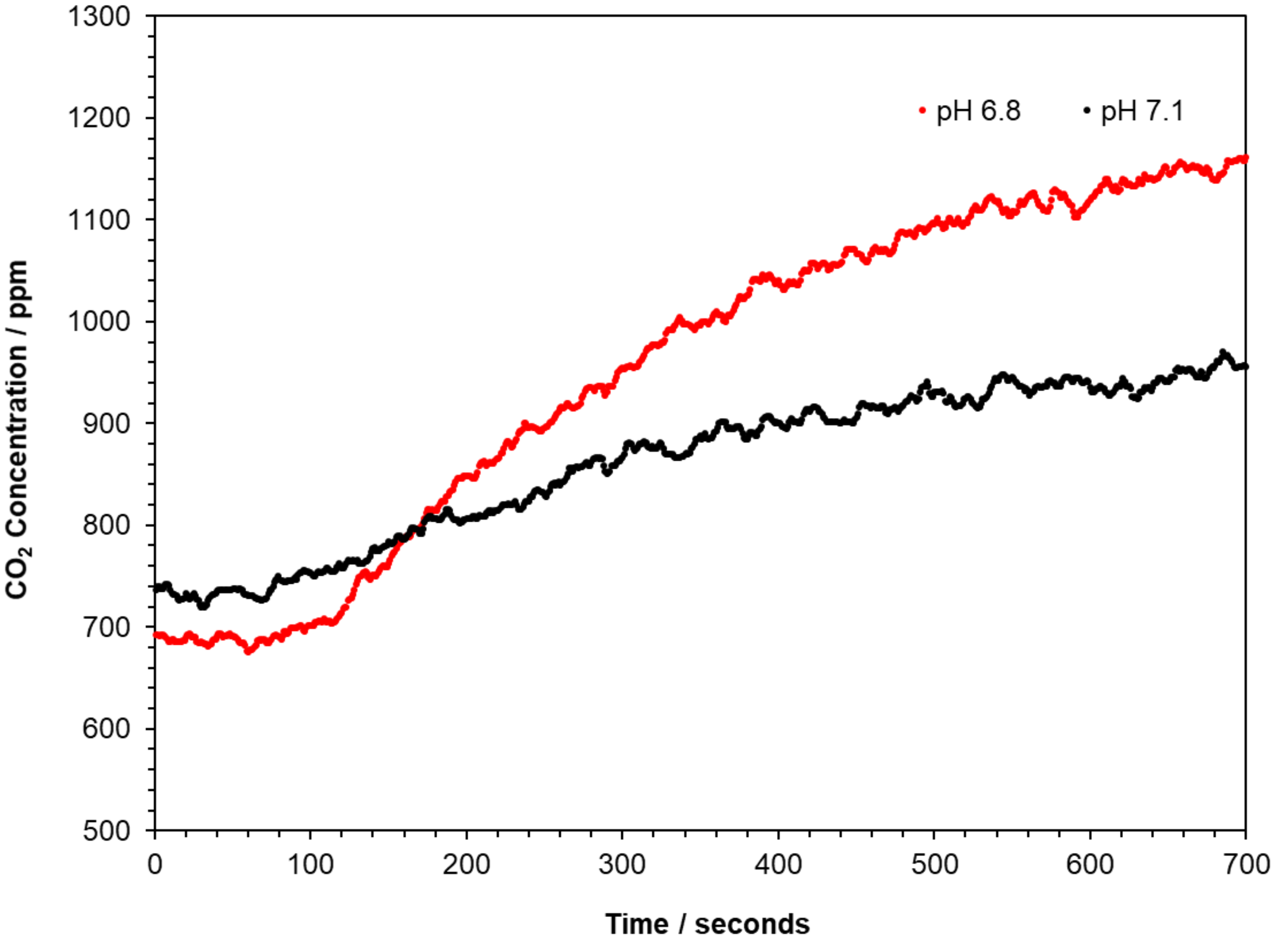

Figure S13| $CO_{2(g)}$ Concentration Increase in an enclosed volume from MEM Nebulisation. Two solutions of MEM, one of pH 6.8 (red line) and one of pH 7.1 (black line) were nebulised into a 7-litre enclosed box, using a Collison nebuliser (50 PSI). Readings from a CO_2 monitor placed within the box are reported here. The time 0 seconds marks the point at which the nebuliser was turned on. The overall increase in CO_2 was found to be a function of the initial pH of the MEM.

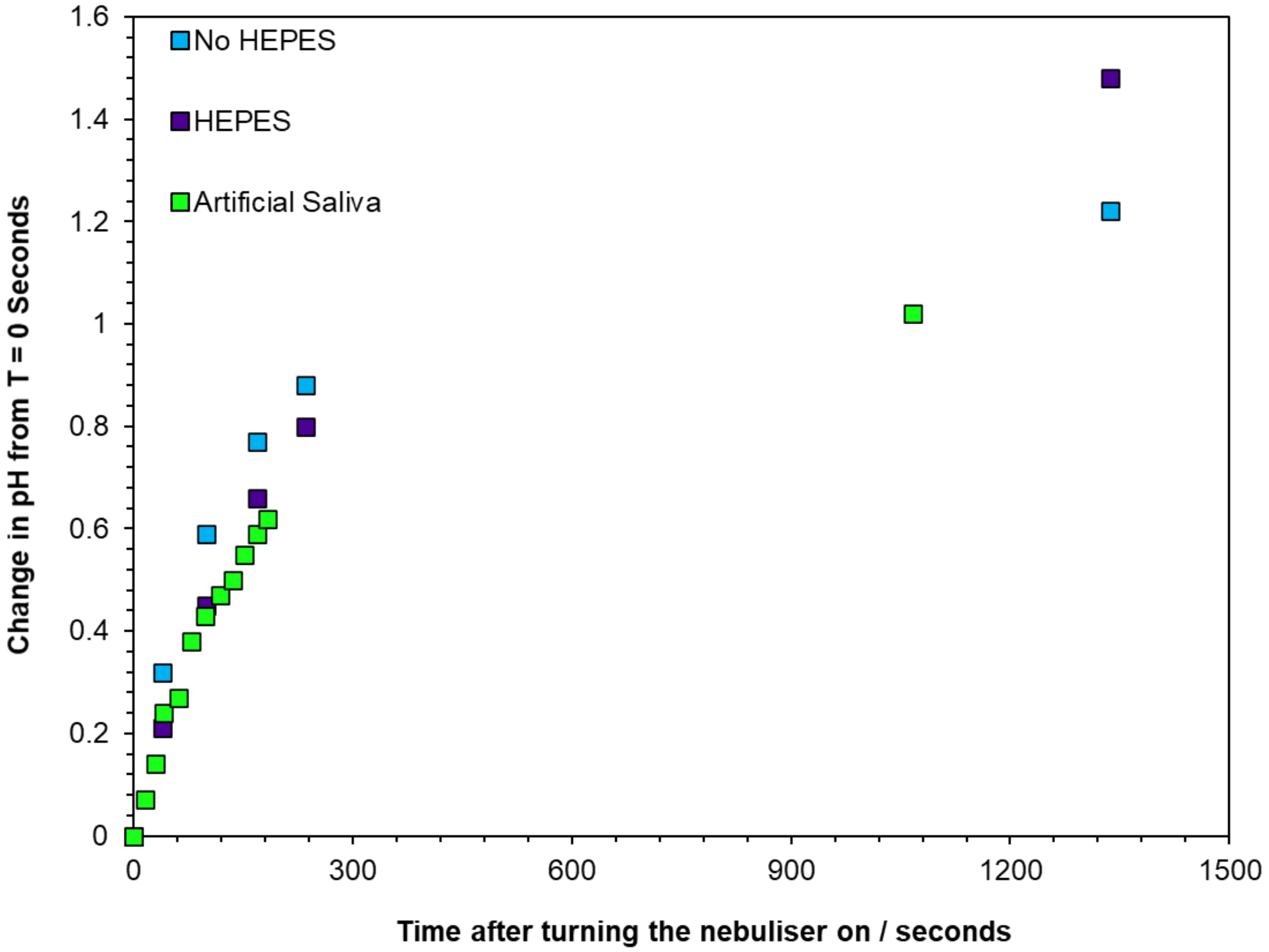

Figure S14| pH Increase in Nebulised Media. Three different solutions were placed into a nebuliser and the pH change in the nebuliser after turning the nebuliser on was measured. DMEM was nebulised both with (purple squares) and without (blue squares) HEPES. Artificial saliva was also nebulised (green squares). pH change is reported as the measured pH at the timepoint minus the pH of the solution before it was loaded into the nebuliser.

SI References

1. S. Matsuyama, *et al.*, Enhanced isolation of SARS-CoV-2 by TMPRSS2- expressing cells. *Proc. Natl. Acad. Sci. U. S. A.* **117**, 7001–7003 (2020).

2. A. D. Davidson, *et al.*, Characterisation of the transcriptome and proteome of SARS-CoV-2 reveals a cell passage induced in-frame deletion of the furin-like cleavage site from the spike glycoprotein. *Genome Med.* **12**, 68 (2020).

3. J. L. Daly, *et al.*, Neuropilin-1 is a host factor for SARS-CoV-2 infection. *Science (80-.).* **370**, 861–865 (2020).

4. L. J. Reed, H. Muench, A Simple Method of Estimating 50 Percent Endpoints. *Am. J. Epidemiol.* **27**, 493–497 (1938).

5. W. J. Glantschnig, S.-H. Chen, Light scattering from water droplets in the geometrical optics approximation. *Appl. Opt.* **20**, 2499 (1981).

6. T. C. Preston, J. P. Reid, Determining the size and refractive index of microspheres using the mode assignments from Mie resonances. *J. Opt. Soc. Am. A* **32**, 2210 (2015).

7. D. A. Hardy, *et al.*, High time resolution measurements of droplet evaporation kinetics and particle crystallisation. *Phys. Chem. Chem. Phys.* **23**, 18568–18579 (2021).
